# Associations between *TNF-α* Polymorphisms and Pneumonia: A Meta-Analysis

**DOI:** 10.1371/journal.pone.0061039

**Published:** 2013-04-08

**Authors:** Li Li, Wei Nie, Weifeng Li, Weifeng Yuan, Wenjie Huang

**Affiliations:** 1 Department of Respiratory Medicine, Guangzhou General Hospital of Guangzhou Military Command, Guangzhou, China; 2 Department of Respiratory Medicine, Shanghai Changzheng Hospital, Second Military Medical University, Shanghai, China; University of California Los Angeles, United States of America

## Abstract

**Background:**

Several studies evaluated the associations of *tumor necrosis factor-α* (*TNF-α*) polymorphisms with pneumonia in different populations. However, the results were conflicting and controversial.

**Methods:**

Databases including PubMed, Embase, Web of Science, and China National Knowledge Infrastructure (CNKI) were searched to find relevant studies. Data were extracted independently by two investigators. Crude odds ratios (ORs) and corresponding 95% confidence intervals (CIs) were estimated.

**Results:**

Twelve case-control studies and one cohort study were included. Overall, no association between *TNF-α* −308A/G polymorphism and pneumonia risk was observed for AA +AG vs. GG (OR = 1.13; 95% CI 0.99–1.30; *P* = 0.07). In addition, *TNF-α* −308A/G polymorphism was not associated with pneumonia mortality (OR = 1.96; 95% CI 0.94–4.09; *P* = 0.07). Furthermore, there was no association of *TNF-α* −238A/G polymorphism with the risk of pneumonia (OR = 1.38; 95% CI 0.84–2.28; *P* = 0.20).

**Conclusions:**

*TNF-α* −308A/G, −238A/G polymorphisms were not associated with pneumonia risk. Moreover, *TNF-α* −308A/G polymorphism did not play a role in the pneumonia mortality risk.

## Introduction

Previous studies evidenced the importance of individual genetic differences on the risk of developing or dying from infection [1]. For example, the familial risk of death from infection has been suggested to be greater than that from either cancer or cardiovascular disease [2]. Pneumonia is a common infectious disease associated with high morbidity and mortality. Therefore, host genetic susceptibility may play a key role in the pathogenesis of pneumonia. So far, a lot of studies have focused on this field, and the *tumor necrosis factor-α* (*TNF-α*) gene has been studied extensively.

TNF-α is a potential proinflammatory cytokine that plays a critical role in inflammatory and immune responses. Excessive TNF-α production contributed to lung injury in a variety of diseases [3], [4] and increased the risk of sepsis [5]. In mechanically ventilated patients, Montón and colleagues found that serum TNF-α level was significantly higher in patients with pneumonia compared with controls [6]. In addition, Bauer et al. showed that levels of serum TNF-α was strongly associated with the degree of lung injury [7]. Furthermore, Puren et al. indicated that plasma levels of TNF-α may be a marker of severity of pneumonia [8]. Collectively, these results suggested that TNF-α might have an important role in the pathophysiology of pneumonia.

*TNF-α* gene is located on chromosome 6, within the class III region of MHC. Several polymorphisms in the promoter region of *TNF-α*, such as −238A/G (rs361525), −308A/G (rs1800629), −857G/A (rs1799724), and −1031T/C (rs1799964), have been identified. These polymorphisms could alter the expression of TNF-α [9]–[11]. Previous studies have assessed the associations between *TNF-α* polymorphisms and the risk and outcomes of pneumonia [12]–[24]. However, the results were inconclusive and remained contradictory. Since most studies only included a modest sample size, each of them might not achieve a reliable conclusion. Meta-analysis is a good method to synthesize data from different studies on the same topic. Therefore, we did this meta-analysis to determine whether *TNF-α* polymorphisms were associated with an increased risk of pneumonia or higher pneumonia mortality. To our knowledge, this was the first meta-analysis of the associations between *TNF-α* polymorphisms and pneumonia risk and mortality.

## Methods

### Publication search

The electronic databases of PubMed, Embase, Web of Science, and China National Knowledge Infrastructure (CNKI) were searched. The following search terms were used: (pneumonia or community-acquired pneumonia or hospital-acquired pneumonia or ventilator-associated pneumonia) and (tumor necrosis factor or TNF or tumor necrosis factor-α or TNF-α) and (polymorphism or mutation or variant). Last search was updated in January 23, 2013. Additional relevant references cited in searched articles were also retrieved. No language restrictions were applied.

### Inclusion and exclusion criteria

The included studies should meet the following criteria: (1) evaluation of the *TNF-α* polymorphisms and risk of pneumonia or pneumonia mortality, (2) using a case-control design or cohort design, (3) sufficient data for estimating an odds ratio (OR) and 95% confidence interval (CI). Studies were excluded if one of the following criteria existed: (1) not relevant to *TNF-α* polymorphisms, TNF-α, pneumonia, or pneumonia mortality, (2) non-clinical studies, (3) reviews or abstracts, and (4) not reported genotype frequencies or numbers. For the overlapping studies, only the one with the largest sample size was included in our study.

### Qualitative assessment

Two investigators (Li and Nie) assessed the quality of each study independently. Any disagreement was resolved by consensus. The predetermined quality assessment criteria were modified from a previous review [25]. This quality scoring system was based on both traditional epidemiologic considerations and genetic issues. Total scores ranged from 0 (worst) to 9 (best) for cohort studies and 0 (worst) to 10 (best) for case-control studies. Case-control studies scoring <5 were defined as low quality, and those ≥5 were defined as high quality. Cohort studies scoring <4 were defined as low quality, and those ≥4 were defined as high quality.

### Data extraction

The following data were collected from each study: the first author's surname, publication year, ethnicity, age group, type of pneumonia, severity of disease, outcome (mortality), sample size, genotyping method, *TNF-α* polymorphisms, and genotype numbers in cases and controls. Two independent reviewers (Li and Nie) collected these data and any discrepancy was resolved by discussion. Authors of the included studies were contacted via E-mail if further study details were needed.

### Statistical analysis

Where the data from at least three similar studies were available, meta-analysis was performed. Thus, we evaluated the strength of associations between *TNF-α* −308A/G, −238A/G polymorphisms and the risk of pneumonia and mortality. For this meta-analysis, we examined the dominant genetic model (AA+AG vs. GG) because data were most commonly either presented in this format or convertible to this format. Crude ORs with 95% CIs were computed to assess the strength of the associations. The statistical significance of OR was determined with *Z* test.

Departure from Hardy-Weinberg equilibrium (HWE) in controls was tested by the Chi-square test. The Q statistic and the *I* ^2^ statistic were used to assess the degree of heterogeneity among the studies included in the meta-analysis. A *P* value greater than 0.10 for the Q-test indicated a lack of heterogeneity among studies, so that the pooled OR estimate of each study was calculated by the fixed-effects model. Otherwise, the random-effects model was used. Subgroup analyses were carried out by ethnicity, type of pneumonia, and severity of disease. Sensitivity analysis was performed by excluding the low quality studies and excluding the study not in HWE. Publication bias was evaluated with the funnel plot and the linear regression asymmetry test by Egger et al. [26] when there were at least 10 studies included in the meta-analysis [27]. All statistical tests were performed by using STATA 11.0 software (Stata Corporation, College Station, TX) and Revman 5.1 software (Nordic Cochrane Center, Copenhagen, Denmark). A *P* value<0.05 was considered statistically significant. Bonferroni correction of critical *P* values was applied when performing a high number of comparisons. In order to avoid spurious positives, the alpha value was lowered to account for the number of comparisons being performed.

## Results

### Study characteristics

**Figure 1:**
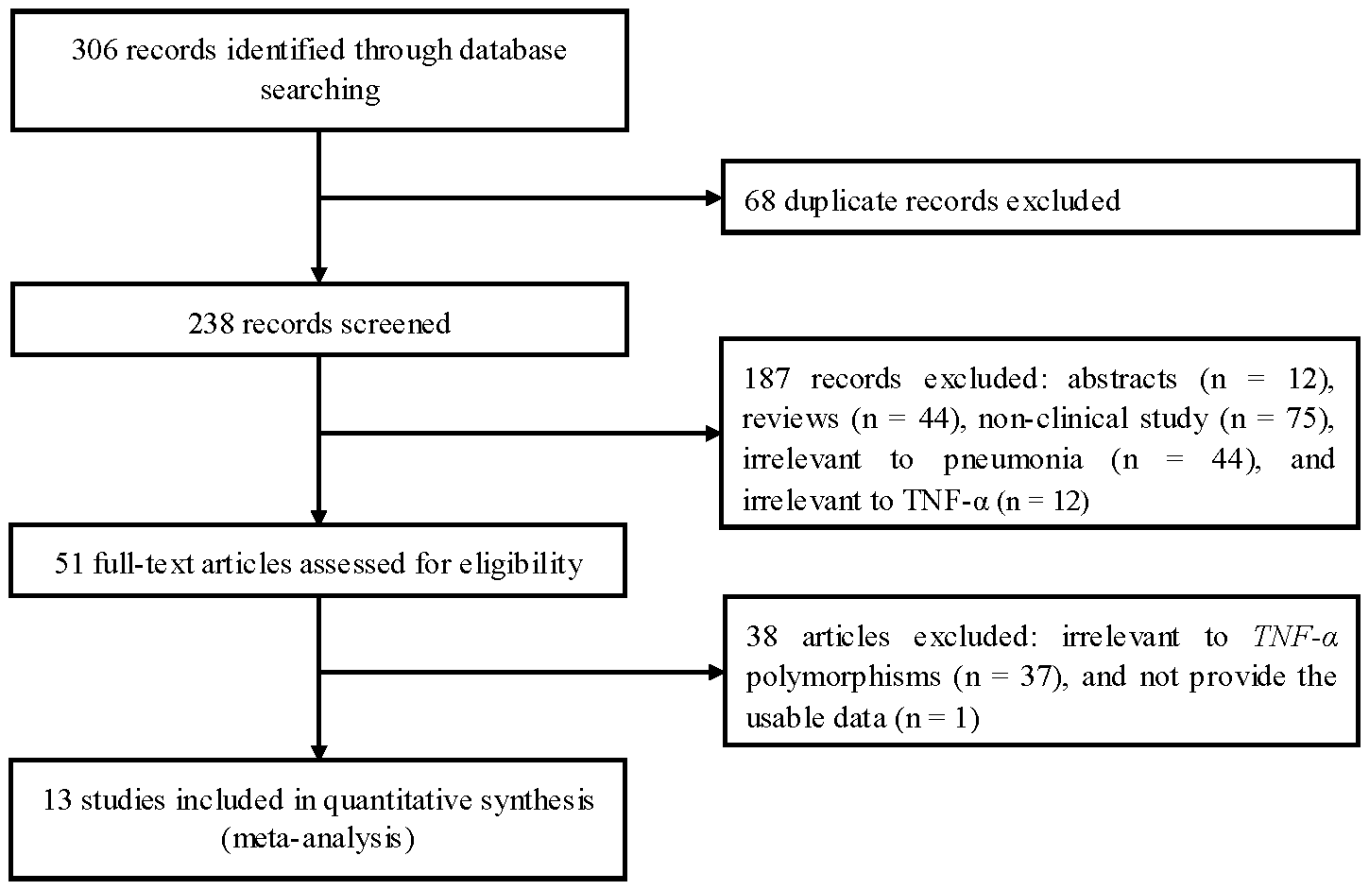
Flow of study identification, inclusion, and exclusion.

Our search strategy yielded 306 articles for review. **Figure 1** shows the literature search and the selection flow chart. Our final pool of eligible studies included 12 case-control studies [13]–[24] and 1 cohort study [12]. **Table 1** presents a summarized description of the 13 ultimately selected articles. Six studies were conducted in Caucasian populations [15], [18], [20]–[22], [24], three in Asian populations [17], [19], [23], and four in mixed ethnic groups [12]–[14], [16]. Most of the studies comprised adult patients [12], [15], [17]–[20], [22]–[24], whereas two studies focused on pediatric patients [13], [14]. Pneumonia was classified as community-acquired pneumonia (CAP) [12], [13], [15], [17], [18], [20], [24], hospital-acquired pneumonia (HAP) [14], [19], ventilator-associated pneumonia (VAP) [22], and mixed pneumonia [16], [23]. Two studies included severe pneumonia patients [14], [22]. Three studies included both severe and non-severe pneumonia patients but the data for these patients could be separately extracted [17], [19], [23]. Seven studies reported pneumonia-related mortality rate [12], [13], [15], [17]–[19], [23]. There were twelve studies on −308A/G [12]–[19], [21]–[24], seven studies on −238A/G [16]–[18], [20]–[23]. No more than two studies on +488A/G, −1031T/C, −863C/A, −857C/T, and −376G/A. The quality scores of studies ranged from 3 to 6. Three studied were defined as low quality studies [14], [16], [17]. One study was not in HWE [24]. Genotype numbers and HWE examination results are presented in **Table 2**.

**Table 1:** Characteristics of the studies included in the meta-analysis.

			Age	Pneumonia	Disease	Mortality	Case	Control	Genotyping		Quality
First author	Year	Ethnicity	group	type	severity	reported	number (n)	number (n)	method	Polymorphisms	score
Waterer [12]	2001	Mixed	Adult	CAP	Mixed	Yes	280	NA	PCR-RFLP	−308A/G	5
Schaaf [13]	2003	Mixed	Pediatric	CAP	NA	Yes	64	50	PCR-RFLP	−308A/G	5
Hedberg [14]	2004	Mixed	Pediatric	HAP	Severe	No	12	161	PCR-RFLP	−308A/G	3
Cipriano [15]	2005	Caucasian	Adult	CAP	Mixed	Yes	20	53	PCR-RFLP	−308A/G	5
Kinder [16]	2007	Mixed	Mixed	Mixed	NA	No	42	217	PCR-RFLP	−308A/G, −238A/G, +488A/G	4
Yuan [17]	2008	Asian	Adult	CAP	Mixed*	Yes	67	50	PCR-RFLP	−308A/G, −238A/G, −1031T/C	3
										−863C/A, −857C/T	
Sole-Violan [18]	2009	Caucasian	Adult	CAP	Mixed	Yes	1136	1152	PCR-RFLP	−308A/G, −238A/G	5
Zhang [19]	2010	Asian	Adult	HAP	Mixed*	Yes	167	110	PCR-RFLP	−308A/G	5
Endeman [20]	2011	Caucasian	Adult	CAP	Mixed	No	200	313	TaqMan	−238A/G	5
Antonopoulou [21]	2012	Caucasian	Mixed	NA	NA	No	27	108	PCR-RFLP	−308A/G, −238A/G, −376G/A	6
Kotsaki [22]	2012	Caucasian	Adult	VAP	Severe	No	213	212	PCR-RFLP	−308A/G, −238A/G, −376G/A	6
Song [23]	2012	Asian	Adult	Mixed	Mixed*	Yes	551	600	Direct sequencing	−308A/G, −238A/G, −857G/A	6
										−1031T/C, −863C/A	
Salnikova [24]	2012	Caucasian	Adult	CAP	NA	No	321	452	PCR	−308A/G	6

* Data for severe pneumonia and non-severe pneumonia patients could be extracted.

CAP, community-acquired pneumonia; HAP, hospital-acquired pneumonia; VAP, ventilator-associated pneumonia; PCR: polymerase chain reaction; RFLP: restriction fragment length polymorphism; NA, not available.

**Table 2:** Distribution of *TNF* −308A/G and −238A/G genotype among cases and controls.

	Case		Control		Hardy-Weinberg
Studies	AA+AG	GG	AA+AG	GG	equilibrium
−308A/G					
Schaaf	20	44	17	33	Yes
Hedberg	3	9	42	111	Yes
Cipriano	8	12	11	42	Yes
Kinder	9	33	63	154	Yes
Yuan	11	56	3	47	Yes
Sole-Violan	280	856	282	870	Yes
Zhang	47	120	20	90	Yes
Antonopoulou	5	22	16	92	Yes
Kotsaki	41	172	29	183	Yes
Song	58	493	40	560	Yes
Salnikova	73	248	105	347	No
−238A/G					
Kinder	11	31	19	198	Yes
Yuan	0	67	1	49	Yes
Sole-Violan	159	976	156	1016	Yes
Endeman	13	187	30	286	Yes
Antonopoulou	7	20	4	104	Yes
Kotsaki	6	207	9	203	Yes
Song	59	492	48	550	Yes

### TNF-α −308A/G polymorphism

**Figure 2:**
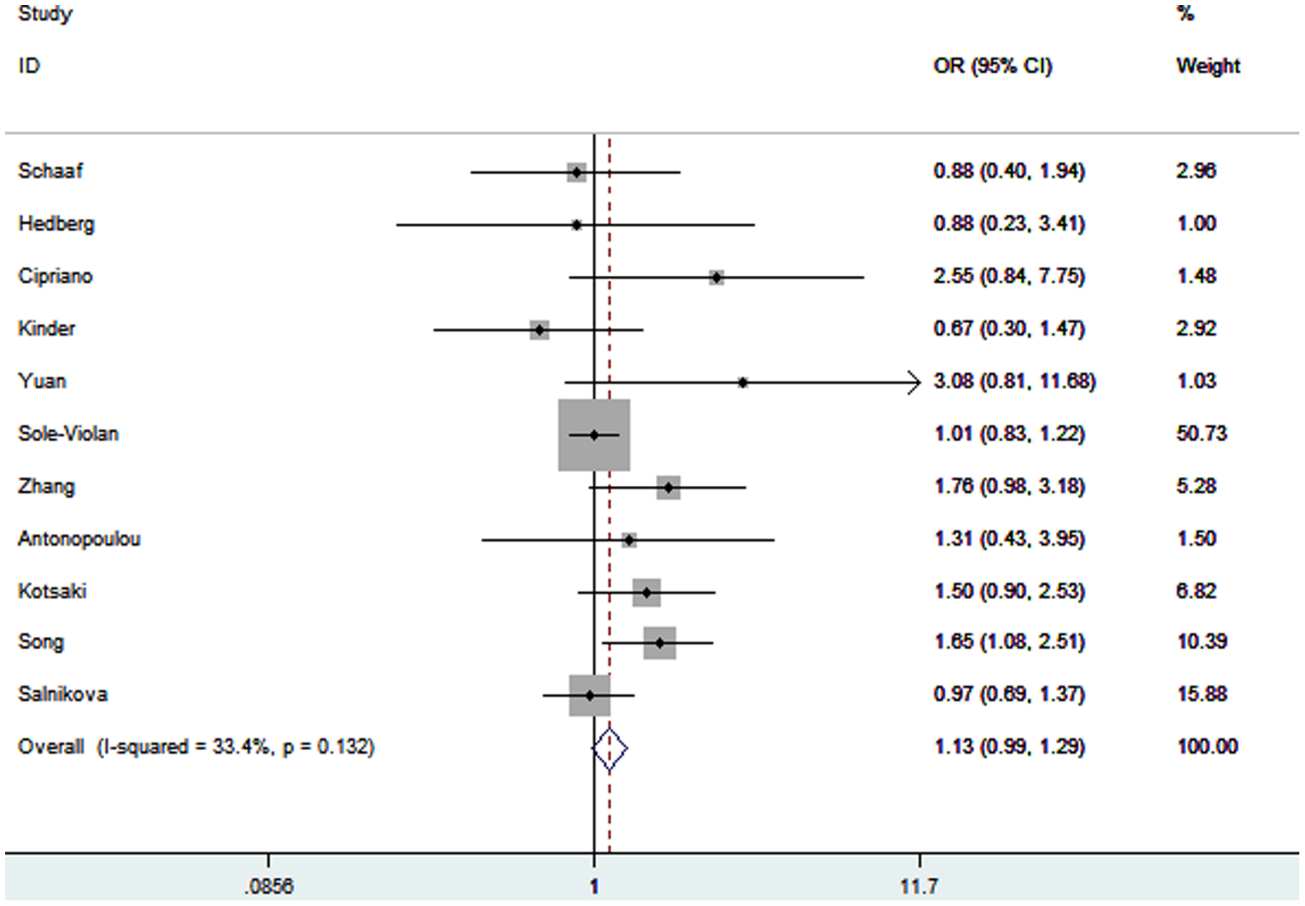
Meta-analysis for the association between pneumonia risk and the *TNF-α* −308A/G polymorphism.

Eleven studies determined the association between *TNF-α* −308A/G polymorphism and pneumonia risk [13]–[19], [21]–[24]. Total sample sizes for case group and control group were 2620 and 3157, respectively. The pooled OR was 1.13 (95% CI 0.99–1.30; *P* = 0.07) (**Figure 2**). In the subgroup analysis by ethnicity, a significant association was found among Asians (OR = 1.76; 95% CI 1.26–2.45; *P* = 0.0008) but not among Caucasians (OR = 1.04; 95% CI 0.89–1.21; *P* = 0.63). Subgroup analysis was also performed by the type of pneumonia. No increased risk was found among CAP patients (OR = 1.03; 95% CI 0.88–1.21; *P* = 0.68). In the subgroup analysis by the severity of pneumonia, significant increase of severe pneumonia risk was found among the A allele carriers (OR = 2.20; 95% CI 1.32–3.66; *P* = 0.002). No significant association was found between *TNF-α* −308A/G polymorphism and non-severe pneumonia risk (OR = 1.24; 95% CI 0.82–1.86; *P* = 0.30).

**Figure 3:**
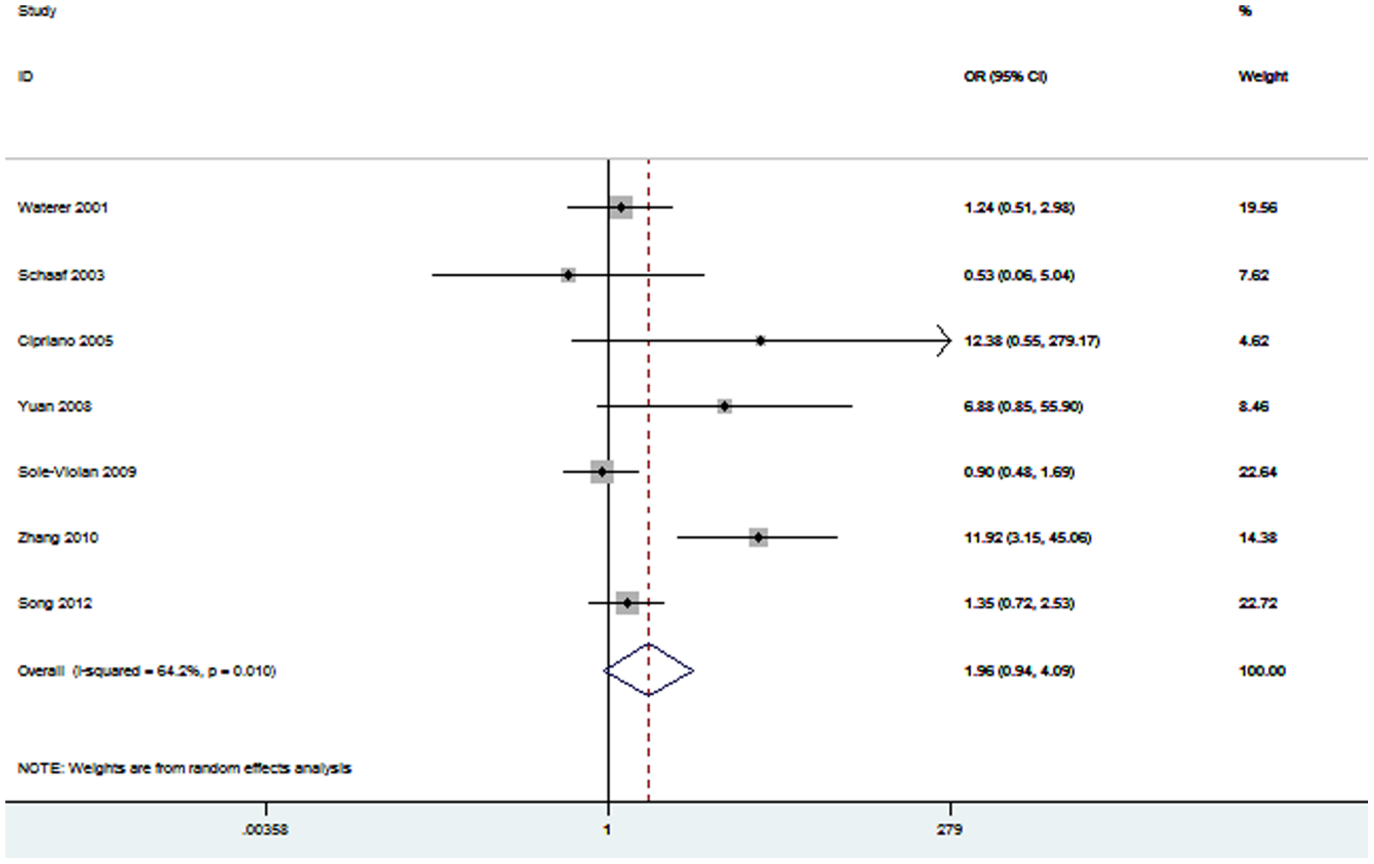
Meta-analysis for the association between mortality risk and the *TNF-α* −308A/G polymorphism.

Seven studies identified the association between *TNF-α* −308A/G polymorphism and pneumonia mortality risk [12], [13], [15], [17]–[19], [23]. Total sample size of patients was 2302. **Figure 3** shows that there is not a significant association between *TNF-α* −308A/G polymorphism and mortality with an OR of 1.96 (95% CI 0.94–4.09; *P* = 0.07). In the subgroup analysis by ethnicity, no association was found among Asians (OR = 4.25; 95% CI 0.85–21.35; *P* = 0.08). Stratification by pneumonia type showed that CAP patients carrying A allele were not associated with mortality risk (OR = 1.15; 95% CI 0.73–1.83; *P* = 0.55). Furthermore, there was no significant association between *TNF-α* −308A/G polymorphism and severe pneumonia mortality risk (OR = 3.45; 95% CI 0.60–19.95; *P* = 0.17). Summary results of comparisons are listed in **Table 3**.

**Table 3:** Summary of meta-analysis results.

		No. of	Test of association				Heterogeneity		
Comparisons	Study	studies	OR (95% CI)	*Z*	*P* Value	Model	*χ* ^2^	*P* Value	*I* ^2^ (%)
−308A/G and pneumonia risk									
AA+AG vs. GG	Overall	11	1.13 (0.99–1.30)	1.82	0.07	F	15.01	0.13	33.0
AA+AG vs. GG	Asian	3	1.76 (1.26–2.45)	3.35	**0.0008**	F	0.77	0.68	0.0
AA+AG vs. GG	Caucasian	5	1.04 (0.89–1.21)	0.63	0.63	F	5.87	0.21	32.0
AA+AG vs. GG	CAP	4	1.05 (0.88–1.26)	0.55	0.58	F	5.28	0.15	43.0
AA+AG vs. GG	Severe	5	2.20 (1.32–3.66)	3.03	**0.002**	R	8.60	0.01	53.0
AA+AG vs. GG	Non-severe	3	1.23 (0.82–1.86)	1.00	0.32	F	0.85	0.65	0.0
AA+AG vs. GG	High quality	8	1.14 (0.99–1.31)	1.87	0.06	F	11.00	0.14	36.0
AA+AG vs. GG	HWE	10	1.17 (1.01–1.35)	2.05	0.04	F	14.12	0.12	36.0
−308A/G and mortality risk									
AA+AG vs. GG	Overall	7	1.96 (0.94–4.09)	1.80	0.07	R	16.74	0.01	64.0
AA+AG vs. GG	Asian	3	4.25 (0.85–21.35)	1.76	0.08	R	9.66	0.008	79.0
AA+AG vs. GG	CAP	5	1.15 (0.73–1.83)	0.60	0.55	F	6.10	0.19	34.0
AA+AG vs. GG	Severe	3	3.45 (0.60–19.95)	1.38	0.17	R	9.78	0.008	80.0
AA+AG vs. GG	High quality	6	1.74 (0.82–3.68)	1.44	0.15	R	14.58	0.01	66.0
−238A/G and pneumonia risk									
AA+AG vs. GG	Overall	7	1.38 (0.84–2.28)	1.27	0.20	R	22.71	0.0009	74.0
AA+AG vs. GG	Caucasian	4	1.21 (0.58–2.54)	0.50	0.62	R	12.87	0.005	77.0
AA+AG vs. GG	CAP	3	0.94 (0.67–1.33)	0.33	0.74	F	2.40	0.30	16.0
AA+AG vs. GG	High quality	5	1.19 (0.74–1.93)	0.72	0.47	R	14.22	0.007	72.0

Bonferroni correction was applied (*P*<0.00294). Bold indicates statistically significant *P* values.

vs., versus; CAP, community-acquired pneumonia; HWE, Hardy-Weinberg equilibrium; R, random-effects model; F, fixed-effects model.

### TNF-α −238A/G polymorphism

**Figure 4:**
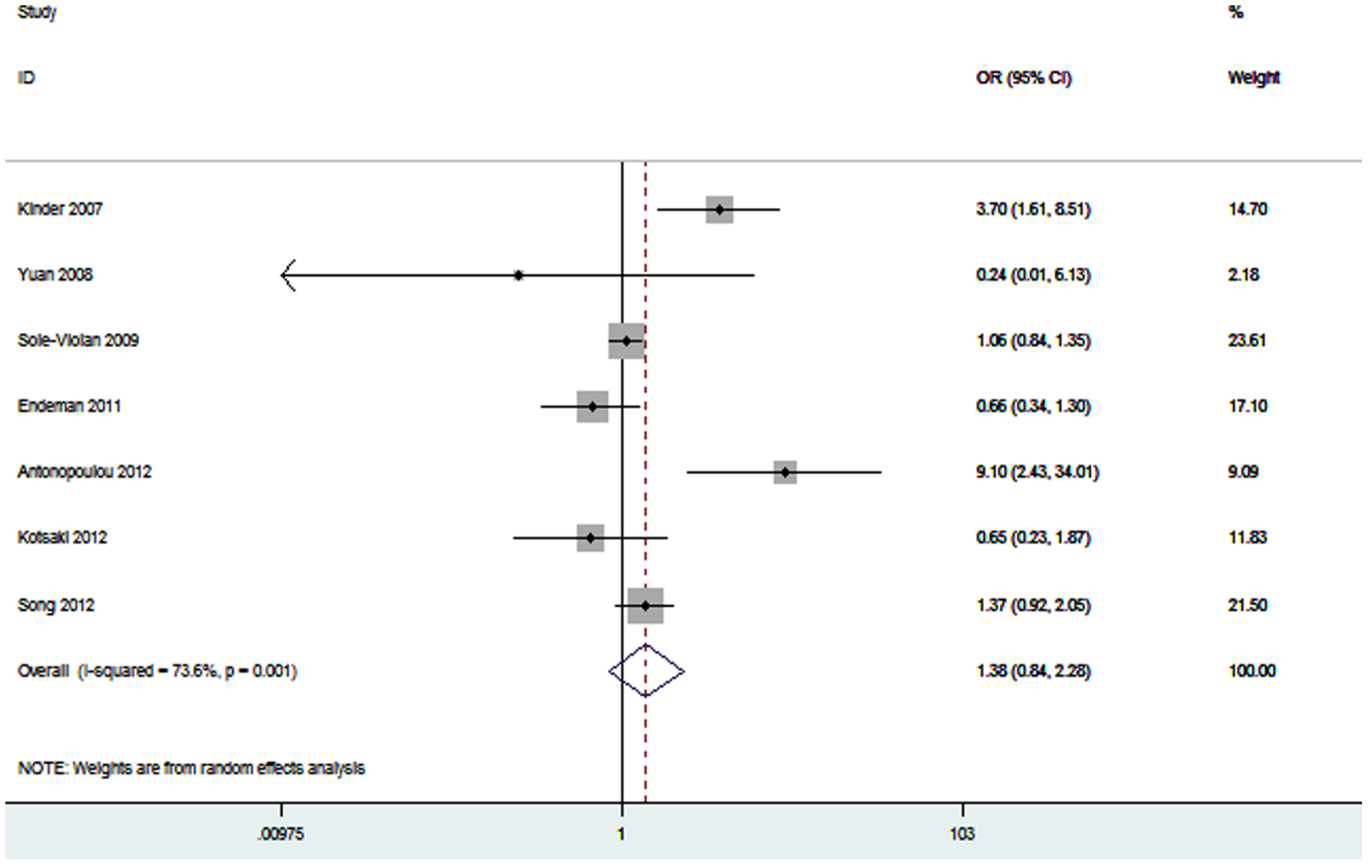
Meta-analysis for the association between pneumonia risk and the *TNF-α* −238A/G polymorphism.

Seven studies (2235 cases and 2673 controls) studied the association between *TNF-α* −238A/G polymorphism and pneumonia risk [16]–[18], [20]–[23]. The pooled OR was 1.38 (95% CI 0.84–2.28, *P* = 0.20) (**Figure 4**). Subgroup analyses performed according to ethnicity and pneumonia type still did not find significant association between −238A/G polymorphism and pneumonia risk (**Table 3**). There were only two studies investigated the association between this polymorphism and mortality risk. Thus, meta-analysis was not performed.

### Sensitivity analysis and Heterogeneity analysis

In order to assess the stability of the results of the meta-analysis, sensitivity analyses were performed by omitting the low quality studies [14], [16], [17] and the HWE-violating study [24], respectively. All the results were not altered which suggested the robustness of our results (**Table 3**).

For *TNF-α* −308A/G polymorphism and pneumonia risk, no significant between-study heterogeneity was observed in the dominant genetic model (*P*_heterogeneity_ = 0.13 and *I^2^* = 33%). For *TNF-α* −308A/G polymorphism and mortality risk, there was statistically significant between-study heterogeneity in the dominant genetic model (*P*_heterogeneity_ = 0.01 and *I^2^* = 64%). In the CAP subgroup, no significant heterogeneity was found (*P*_heterogeneity_ = 0.19 and *I^2^* = 34%). For *TNF-α* −238A/G polymorphism and pneumonia risk, there was also statistically significant between-study heterogeneity (*P*_heterogeneity_ = 0.0009 and *I^2^* = 74%). As shown in **Table 3**, the heterogeneity was significantly decreased in the CAP subgroup (*P*_heterogeneity_ = 0.30 and *I^2^* = 16%).

### Publication bias

**Figure 5:**
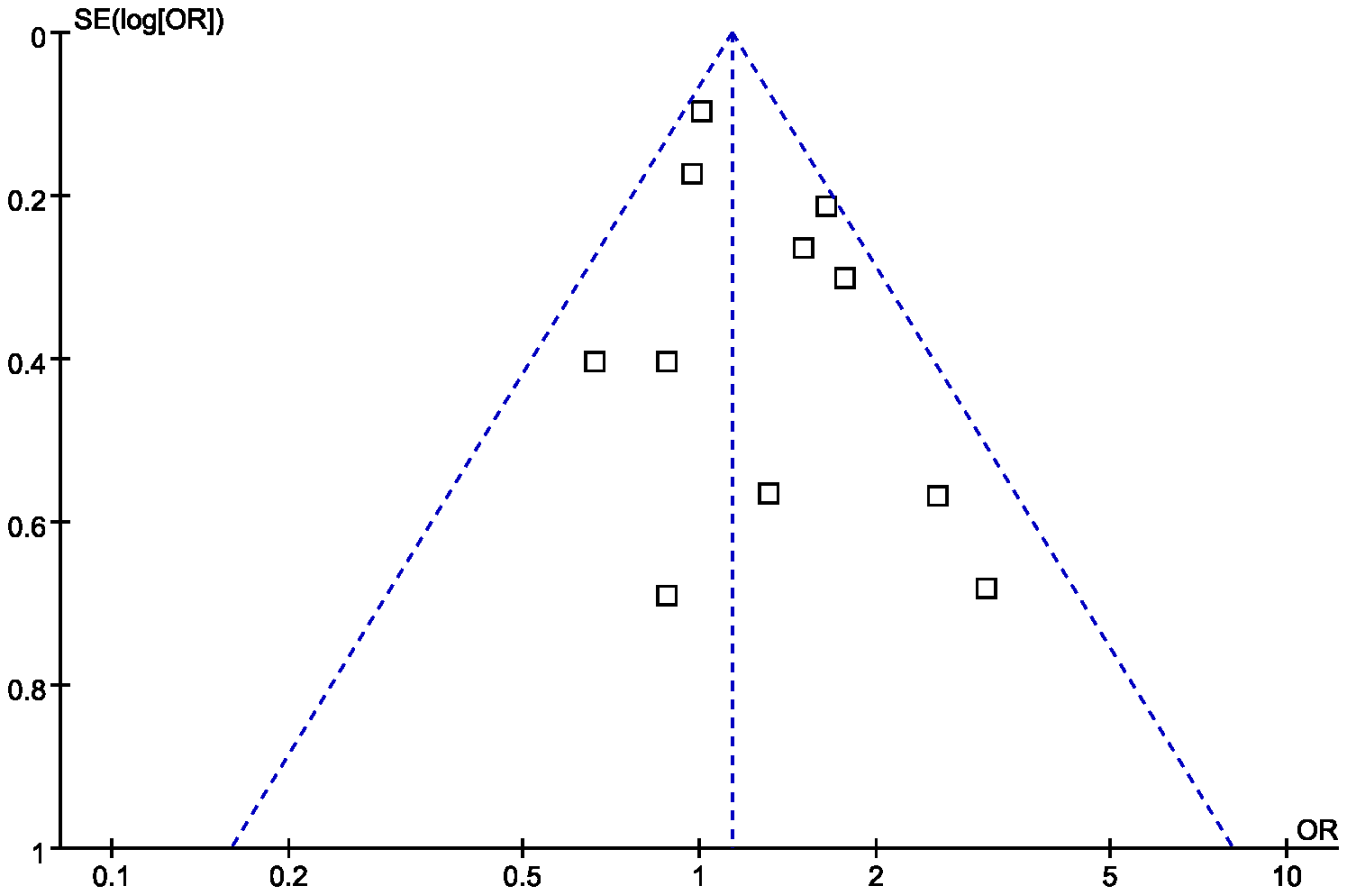
Funnel plot for publication bias in the association between pneumonia risk and the *TNF-α* −308A/G polymorphism.

Publication bias was examined by funnel plot qualitatively and estimated by Egger's test quantitatively. The shape of the funnel plot seemed almost symmetrical (**Figure 5**). Egger's test did not show evidence of publication bias (*P* = 0.191).

## Discussion

This meta-analysis of 13 studies systematically evaluated the associations between *TNF-α* −308A/G, −238A/G polymorphisms and risk and mortality of pneumonia. We found that −308A/G polymorphism was not a risk factor for developing pneumonia in the overall study population. In the subgroup analysis, however, we noted that individuals carrying A allele had increased pneumonia risk in Asians, but not in Caucasians. This result indicated that interactions between different ethnicities and genetic variants may contribute to pneumonia risk. We also carried out subgroup analysis for type of pneumonia. No evidence supported the association between this polymorphism and risk of CAP. However, we found that patients with A allele had increased severe pneumonia risk, suggesting a possible role of −308A/G polymorphism in severe pneumonia pathogenesis. Although subgroup analyses found significant associations among Asians and patients with severe pneumonia, we should exercise great caution in interpreting apparent results. Chance is the explanation for spurious subgroup analyses. Thus, these results should be validated by future studies.

A non-significant result was observed for the association of −308A/G polymorphism with pneumonia-related mortality. Teuffel and colleagues also did not find an association between this polymorphism and mortality from sepsis in their meta-analysis [5]. When stratified for ethnicity, type of pneumonia, and disease severity, no increased risk for fatal outcome was found among Asians, CAP patients, and severe pneumonia patients. There were no more than 2 studies using Caucasians evaluated the association between −308A/G polymorphism and mortality risk. The positive association between this condition and mortality risk could not be ruled out, because studies with small sample size may have insufficient statistical power to detect a slight effect. Therefore, more studies with Caucasian population are needed to assess the effect of −308A/G polymorphism on pneumonia mortality risk.

Results from our meta-analysis showed the lack of association between −238A/G polymorphism and pneumonia risk. Subgroup analyses also did not detect significant association between this polymorphism and pneumonia risk. These results suggested that −238A/G polymorphism might have no role in the etiology of pneumonia. However, the study from Pappachan et al. [28] showed a significant association between the −238A/G polymorphism and mortality from systemic inflammatory response syndrome (SIRS). They found that −238A was significantly higher in patients who died in ICU compared to those who survived [28]. Thus, *TNF-α* −238A/G polymorphism may play a possible role in the pneumonia-related mortality. However, there were only two studies estimated this association and meta-analysis could not be conducted. This issue is needed to be further studied.

Pneumonia is a complex inflammatory disease. Moderate degree of local inflammation is required to control infection; however, excessive release of inflammatory cytokines favours intracellular and extracellular bacterial growth and organ dysfunction [29], [30]. Lee et al. [31] found that TNF-α augmented *Escherichia coli* growth both *in vitro* and *in vivo*. In addition, systemic administration of TNF-α in rat worsened pneumonia by reducing alveolar neutrophil recruitment and bacterial clearance [32], [33]. Moreover, Yende et al. [34] indicated that preinfection systemic level of TNF-α was associated with higher risk of CAP requiring hospitalization. Therefore, TNF-α may play a pivotal role in the development of pneumonia. Early study demonstrated that a functional mutation (−308A/G) in the *TNF-α* gene could influence the expression of TNF-α [35]. The presence of the A substitution led to increase in the binding of nuclear factors and enhanced transcription of the gene [36]. Recently, Song et al. [23] showed severe sepsis patients with AA+AG genotypes had significantly higher TNF-α serum concentrations than those with GG genotype. Therefore, it was biologically plausible that −308A/G polymorphism which could affect TNF-α level might influence the susceptibility to pneumonia and mortality risk. However, negative results were found in our study. There could be two potential reasons for the results. First, because of the complex nature of pneumonia, it was unlikely that a single nucleotide polymorphism in one single gene would be obviously associated with an increased pneumonia or mortality risk, without consideration of any other polymorphic susceptible genes. Second, other factors, such as age, pathogenic organism, medical treatment, and nutrient status etc., can also influence the development or the prognosis of pneumonia.

Kaluza et al. [37] found that the psoriasis-associated *TNF-α* −238A showed a significantly decreased transcriptional activity. Furthermore, Huizinga and coworkers revealed that the multiple sclerosis patients with −238 GA produced lower TNF-α compared to those with −238 GG [9]. Nevertheless, no published studies analyzed the relationship between −238A/G polymorphism and TNF-α production in pneumonia patients. Thus, whether −238A/G polymorphism influenced the pneumonia risk was still unclear. The functional studies of −238A/G polymorphism in pneumonia are required.

We should address the importance of heterogeneity and publication bias, which might influence the results of meta-analysis. In our meta-analysis, significant heterogeneity was observed in −308A/G and mortality risk and also in −238A/G and pneumonia risk. Subgroup analysis was used to explore the sources of heterogeneity. After subgroup analysis by the the type of pneumonia, the heterogeneity was effectively decreased in the CAP subgroup. Therefore, it could be speculated that the large heterogeneity mainly resulted from the type of pneumonia. In addition, funnel plot and Egger's test were performed to find potential publication bias. No significant publication bias was noted. Furthermore, we carried out the sensitivity analysis. Removal of the low quality studies or the HWE-violating study did not change the conclusions of meta-analysis. These procedures suggested that our results were stable and robust.

Several limitations of this meta-analysis should be considered when interpreting results. First, the number of included studies was moderate. It was possible that some relevant published studies or unpublished studies with negative results were missed. Second, most of the studies were conducted in Caucasian and Asian populations. Therefore, our results may be applicable only to these ethnic groups. Third, because insufficient data could be extracted from the primary publications, we could not assess the effects of +488A/G, −1031T/C, −863C/A, −857C/T, and −376G/A polymorphisms on pneumonia risk and mortality risk. Fourth, the overall outcome was based on unadjusted data, whereas a more precise analysis could be performed if individual data were available to allow adjustment. Finally, pneumonia is a complex disease, and many genes are related to pneumonia [38]. However, we could not address gene-gene interactions in our study due to the lack of the related information.

In conclusion, our results suggested that *TNF-α* −308A/G polymorphism and −238A/G polymorphism were not associated with the risk of pneumonia in the overall population. In addition, *TNF-α* −308A/G polymorphism was not associated with pneumonia mortality. Future large sample size studies with more ethnic groups are needed to confirm our findings. Moreover, other *TNF-α* polymorphisms and gene-gene interactions should also be considered in future studies.

## References

[1] Waterer GW, Bruns AHW (2010) Genetic risk of acute pulmonary infections and sepsis. Expert Rev Respir Med 4: 229–238. 20406089 10.1586/ers.10.13

[2] Sørensen TIA, Nielsen GG, Andersen PK, Teasdale TW (1988) Genetic and environmental influences on premature death in adult adoptees. N Engl J Med 318: 727–732. 3347221 10.1056/NEJM198803243181202

[3] Wright TW, Pryhuber GS, Chess PR, Wang Z, Notter RH, et al (2004) TNF receptor signaling contributes to chemokine secretion, inflammation, and respiratory deficits during Pneumocystis pneumonia. J Immunol 172: 2511–2521. 14764724 10.4049/jimmunol.172.4.2511

[4] Hildebrandt GC, Olkiewicz KM, Corrion LA, Chang Y, Clouthier SG, et al (2004) Donor-derived TNF-α regulates pulmonary chemokine expression and the development of idiopathic pneumonia syndrome after allogeneic bone marrow transplantation. Blood 104: 586–593. 15069018 10.1182/blood-2003-12-4259

[5] Teuffel O, Ethier MC, Beyene J, Sung L (2010) Association between tumor necrosis factor-[alpha] promoter-308 A/G polymorphism and susceptibility to sepsis and sepsis mortality: A systematic review and meta-analysis. Crit Care Med 38: 276–282. 19789454 10.1097/CCM.0b013e3181b42af0

[6] Montón C, Torres A, El-Ebiary M, Filella X, Xaubet A, et al (1999) Cytokine expression in severe pneumonia: a bronchoalveolar lavage study. Crit Care Med 27: 1745–1753. 10507593 10.1097/00003246-199909000-00008

[7] Bauer TT, Monton C, Torres A, Cabello H, Fillela X, et al (2000) Comparison of systemic cytokine levels in patients with acute respiratory distress syndrome, severe pneumonia, and controls. Thorax 55: 46–52. 10607801 10.1136/thorax.55.1.46 PMC1745592

[8] Puren AJ, Feldman C, Savage N, Becker PJ, Smith C (1995) Patterns of cytokine expression in community-acquired pneumonia. Chest 107: 1342–1349. 7750329 10.1378/chest.107.5.1342

[9] Huizinga TWJ, Westendorp RGJ, Bollen ELEM, Keijsers V, Brinkman B, et al (1997) TNF-α promoter polymorphisms, production and susceptibility to multiple sclerosis in different groups of patients. J Neuroimmunol 72: 149–153. 9042107 10.1016/s0165-5728(96)00182-8

[10] Hellmig S, Fischbach W, Goebeler-Kolve ME, Fölsch UR, Hampe J, et al (2005) A functional promotor polymorphism of TNF-α is associated with primary gastric B-cell lymphoma. Am J Gastroenterol 100: 2644–2649. 16393214 10.1111/j.1572-0241.2005.00338.x

[11] Lindholm E, Bakhtadze E, Cilio C, Agardh E, Groop L, et al (2008) Association between LTA, TNF and AGER polymorphisms and late diabetic complications. PLoS ONE 3: e2546. 18575614 10.1371/journal.pone.0002546 PMC2429972

[12] Waterer GW, Quasney MW, Cantor RM, Wunderink RG (2001) Septic shock and respiratory failure in community-acquired pneumonia have different TNF polymorphism associations. Am J Respir Crit Care Med 163: 1599–1604. 11401880 10.1164/ajrccm.163.7.2011088

[13] Schaaf BM, Boehmke F, Esnaashari H, Seitzer U, Kothe H, et al (2003) Pneumococcal septic shock is associated with the interleukin-10-1082 gene promoter polymorphism. Am J Respir Crit Care Med 168: 476–480. 12746253 10.1164/rccm.200210-1164OC

[14] Hedberg CL, Adcock K, Martin J, Loggins J, Kruger TE, et al (2004) Tumor Necrosis Factor [alpha]-308 Polymorphism Associated With Increased Sepsis Mortality in Ventilated Very Low Birth Weight Infants. Pediatr Infect Dis J 23: 424–428. 15131465 10.1097/01.inf.0000122607.73324.20

[15] Cipriano C, Caruso C, Lio D, Giacconi R, Malavolta M, et al (2005) The −308G/A polymorphism of TNF-α influences immunological parameters in old subjects affected by infectious diseases. Int J Immunogenet 32: 13–18. 15686588 10.1111/j.1744-313X.2005.00490.x

[16] Kinder BW, Freemer MM, King TE Jr, Lum RF, Nititham J, et al (2007) Clinical and genetic risk factors for pneumonia in systemic lupus erythematosus. Arthritis Rheum 56: 2679–2686. 17665457 10.1002/art.22804 PMC2875177

[17] Yuan WF, Huang WJ, Liang K (2008) Single nucleotide polymorphisms of tumor necrosis factor-alpha gene are associated with severe adult community acqnired pneumonia in Chinese. Chinese J Pathophysiology 24: 2391–2395.

[18] Sole-Violan J, de Castro F, Garcia-Laorden MI, Blanquer J, Aspa J, et al (2010) Genetic variability in the severity and outcome of community-acquired pneumonia. Respir Med 104: 440–447. 19900796 10.1016/j.rmed.2009.10.009

[19] Zhang DZ, Zhou WC, Rao GL, Fan LM, Wu XY (2010) Relationship Between Tumor Necrosis Factor-α Gene Promoter Region in −308 Site Polymorphism and Hospital-acquired Pneumonia. Chin J Nosocomiol 20: 929–931.

[20] Endeman H, Meijvis S, Rijkers G, van Velzen-Blad H, Van Moorsel C, et al (2011) Systemic cytokine response in patients with community-acquired pneumonia. Eur Respir J 37: 1431–1438. 20884746 10.1183/09031936.00074410

[21] Antonopoulou A, Baziaka F, Tsaganos T, Raftogiannis M, Koutoukas P, et al (2012) Role of tumor necrosis factor gene single nucleotide polymorphisms in the natural course of 2009 influenza A H1N1 virus infection. Int J Infect Dis 16: e204–e208. 22269998 10.1016/j.ijid.2011.11.012

[22] Kotsaki A, Raftogiannis M, Routsi C, Baziaka F, Kotanidou A, et al (2012) Genetic polymorphisms within tumor necrosis factor gene promoter region: A role for susceptibility to ventilator-associated pneumonia. Cytokine 59: 358–363. 22609212 10.1016/j.cyto.2012.04.040

[23] Song Z, Song Y, Yin J, Shen Y, Yao C, et al (2012) Genetic Variation in the TNF Gene Is Associated with Susceptibility to Severe Sepsis, but Not with Mortality. PLoS ONE 7: e46113. 23029405 10.1371/journal.pone.0046113 PMC3459853

[24] Salnikova LE, Smelaya TV, Moroz VV, Golubev AM, Rubanovich AV (2012) Host genetic risk factors for community-acquired pneumonia. Gene [In Press]. 10.1016/j.gene.2012.10.027 23107763

[25] Clark MF, Baudouin SV (2006) A systematic review of the quality of genetic association studies in human sepsis. Intensive Care Med 32: 1706–1712. 16957907 10.1007/s00134-006-0327-y

[26] Egger M, Smith GD, Schneider M, Minder C (1997) Bias in meta-analysis detected by a simple, graphical test. BMJ 315: 629–634. 9310563 10.1136/bmj.315.7109.629 PMC2127453

[27] Higgins JPT, Green S (2011) Cochrane Handbook for Systematic Reviews of Interventions Version 5.1.0. [updated March 2011]. The Cochrane Collaboration. Available from www.cochrane-handbook.org.

[28] Pappachan JV, Coulson TG, Child NJA, Markham DJ, Nour SM, et al (2009) Mortality in adult intensive care patients with severe systemic inflammatory response syndromes is strongly associated with the hypo-immune TNF-238A polymorphism. Immunogenetics 61: 657–662. 19714324 10.1007/s00251-009-0395-6

[29] Confalonieri M, Meduri GU (2011) Glucocorticoid treatment in community-acquired pneumonia. Lancet 377: 1982–1984. 21636121 10.1016/S0140-6736(11)60777-0

[30] Hotchkiss RS, Karl IE (2003) The pathophysiology and treatment of sepsis. N Engl J Med 348: 138–150. 12519925 10.1056/NEJMra021333

[31] Lee JH, Del Sorbo L, Khine AA, de Azavedo J, Low DE, et al (2003) Modulation of bacterial growth by tumor necrosis factor-α in vitro and in vivo. Am J Respir Crit Care Med 168: 1462–1470. 12958055 10.1164/rccm.200302-303OC

[32] White JC, Nelson S, Winkelstein JA, Booth FVML, Jakab GJ (1986) Impairment of antibacterial defense mechanisms of the lung by extrapulmonary infection. J Infect Dis 153: 202–208. 3511157 10.1093/infdis/153.2.202

[33] Mason CM, Dobard E, Summer WR, Nelson S (1997) Intraportal lipopolysaccharide suppresses pulmonary antibacterial defense mechanisms. J Infect Dis 176: 1293–1302. 9359731 10.1086/514125

[34] Yende S, Tuomanen EI, Wunderink R, Kanaya A, Newman AB, et al (2005) Preinfection systemic inflammatory markers and risk of hospitalization due to pneumonia. Am J Respir Crit Care Med 172: 1440–1446. 16166617 10.1164/rccm.200506-888OC PMC2718438

[35] Wilson AG, Symons JA, McDowell TL, McDevitt HO, Duff GW (1997) Effects of a polymorphism in the human tumor necrosis factor α promoter on transcriptional activation. Proc Natl Acad Sci U S A 94: 3195–3199. 9096369 10.1073/pnas.94.7.3195 PMC20345

[36] Kroeger KM, Carville KS, Abraham LJ (1997) The −308 tumor necrosis factor-α promoter polymorphism effects transcription. Mol Immunol 34: 391–399. 9293772 10.1016/s0161-5890(97)00052-7

[37] Kaluza W, Reuss E, Grossmann S, Hug R, Schopf RE, et al (2000) Different transcriptional activity and in vitro TNF-α production in psoriasis patients carrying the TNF-α 238A promoter polymorphism. J Invest Dermatol 114: 1180–1183. 10844563 10.1046/j.1523-1747.2000.00001.x

[38] Waterer GW (2012) Community-Acquired Pneumonia: Genomics, Epigenomics, Transcriptomics, Proteomics, and Metabolomics. Semin Respir Crit Care Med 33: 257–265. 22718211 10.1055/s-0032-1315637

